# Supplementary material for: Evolutionary history of a Scottish harbour seal population
Source: PeerJ. 2020 Jul 10;8:e9167. doi: 10.7717/peerj.9167 (PMC7357561; doi:10.7717/peerj.9167)
Supplement: Supplemental Information 1 [file peerj-08-9167-s001.docx]

**Title page:** Nikolic, N., Thompson, P., de Bruyn, M., Macé, M., Chevalet, C. Evolutionary history of a Scottish harbour seal population.

**Appendix A.** Potential dinucleotide microsatellite markers considered in this study.

| **Species** | **Markers** | **Tested** | **Not tested because** | **Amplification** |
| --- | --- | --- | --- | --- |
| ***Phoca vitulina*** | SGPV3 | yes |  | ok |
|  | SGPV10 | yes |  | ok |
|  | SGPV11 | yes |  | ok |
|  | SGPV16 | yes |  | ok |
|  | SGPV17 | yes |  | ok |
|  | SGPV9 (=GS8) | yes |  | ok |
|  | SGPV2 | no | Complex pattern: (TC)4(TGTCTC)3(TC)9(AC)22 |  |
|  | PVC19 | yes |  | ok |
|  | PVC26 | yes |  | ok |
|  | PVC29 | yes |  | ok |
|  | PVC30 | yes |  | ok |
|  | PVC63 | yes |  | ok |
|  | PVC74 | yes |  | ok |
|  | PVC78 | yes |  | ok |
|  | Pvc3 | no | Complex pattern |  |
|  | Pvc4 | no | Complex pattern |  |
|  | Pvc5 | yes |  | nothing |
|  | Pvc7 | no | Complex pattern |  |
|  | Pvc9 | no | Complex pattern |  |
|  | Pvc18 | no | Complex pattern |  |
|  | Pvc23 | yes |  | nothing |
| ***Halichoerus grypus*** | GS7 | yes |  | ok |
|  | GS1 | yes |  | ok |
|  | GS2 | yes |  | ok |
|  | GS3 | yes |  | ok |
|  | GS4 | yes |  | ok |
| ***Hydrurga leptonyx*** | H12 | yes |  | ok |
|  | Hl20 | yes |  | ok |
|  | Hl16 | yes |  | ok |
|  | Hl15 | yes |  | ok |
|  | H14 | yes |  | nothing |
| ***Odobenus rosmarus rosmarus*** | OrrFCB23 | yes |  | ok |
|  | OrrFCB2 | yes |  | ok |
|  | OrrFCB1 | yes |  | ok |
|  | OrrFCB24 | yes |  | ok |
|  | OrrFCB11 | yes |  | nothing |

**Appendix B.** Characteristics of 25 microsatellite loci and measures of information content derived from harbour seals of the Moray Firth. Tm° C (annealing temperature); Size range (minimum and maximum allele sizes); A (number of alleles on overall individuals). *Redesigned primers in bold. Two last columns contain information on fluorescent labelling and pool number for multiplex PCRs.

| **Source species** | **Locus names** | **Genbank names** | **Primers** | **Tm° C** | **References** | **Size range** | **A** | **Labels** | **Pool** |
| --- | --- | --- | --- | --- | --- | --- | --- | --- | --- |
| *Phoca vitulina* | SGPv9 | G02096 | TAGTGTTTGGAAATGAGTTGGCA | 55 | Allen et al. 1995 | 95-169 | 3 | Ned | 1 |
|  | (or GS8) |  | ACTGATCCTTGTGAATCCCAGC |  |  |  |  |  |  |
|  | SGPV11 | U65444 | GTGCTGGTGAATTAGCCCATTATAAG | 55 | Goodman 1997 | 147-161 | 3 | Ned | 2 |
|  |  |  | CAGAGTAAGCACCCAAGGAGCAG |  |  |  |  |  |  |
|  | SGPV17 | U65446 | TTAACAACTCCATTATCATTTGAGCC | 50 | Goodman 1997 | 153-161 | 3 | Ned | 3 |
|  |  |  | CTGGTGTGTTAGTGAGGGTTCTGC |  |  |  |  |  |  |
|  | PVC63 | L40985 | **CCTGGACTTTGTTTATACCT*** | 55 | Coltman et al. 1996 | 101 | 1 | Ned | 4 |
|  |  |  | GCATGAGTTCATCTAGGGA |  |  |  |  |  |  |
|  | PVC74 | L40984 | CCATCTGTGTCCTCTGATAG | 50 | Coltman et al. 1996 | 126 | 1 | Ned | 5 |
|  |  |  | CTGATATTCCATGTCTGAGATA |  |  |  |  |  |  |
|  | SGPV10 | U65443 | TCATGAATTGGTATTAGACAAAG | 55 | Goodman 1997 | 135-137 | 2 | Hex | 1 |
|  |  |  | TTCACTTAGCATAATTCCCTC |  |  |  |  |  |  |
|  | SGPV16 | U65445 | TCTGAGAGATTCAGAGTAACCTTC | 55 | Goodman 1997 | 250-280 | 14 | Hex | 2 |
|  |  |  | AGCTAGTGTTAATGATGGTGTG |  |  |  |  |  |  |
|  | SGPV3 | U65442 | GCAGACAACACCAAGAATGAACCC | 55 | Goodman 1997 | 124 | 1 | Hex | 3 |
|  |  |  | ACATCAACATTCTCAGTATGGGTGG |  |  |  |  |  |  |
|  | PVC19 | L40989 | GGGTGAACAGGATTTATCC | 45 | Coltman et al. 1996 | 96-104 | 2 | Hex | 5 |
|  |  |  | GTGCTAGATAACAATCCTAC |  |  |  |  |  |  |
|  | PVC26 | L40988 | ATTTTCTCCATACCTACATAAT | 44 | Coltman et al. 1996 | 116 | 1 | Hex | 6 |
|  |  |  | ATTGTGATCCCATTTTTGTAA |  |  |  |  |  |  |
|  | PVC29 | L40987 | AATTGTGTTGTTTACATCTC | 45 | Coltman et al. 1996 | 101 | 1 | Hex | 7 |
|  |  |  | AACCAGAAGAATAGAATTTGCAT |  |  |  |  |  |  |
|  | PVC30 | L40986 | GCATGTGATCTTACAGCAAT | 45 | Coltman et al. 1996 | 172-181 | 4 | Hex | 8 |
|  |  |  | CATGGGTTCTCAATAGAAGA |  |  |  |  |  |  |
|  | PVC78 | L40983 | GAGTATACCTCCATACTACAC | 52 | Coltman et al. 1996 | 130-179 | 4 | Hex | 9 |
|  |  |  | AGTTGTTCTCCTGACCCAAG |  |  |  |  |  |  |
| *Halichoerus grypus* | GS7 | G02095 | ACCTGCCATAGTGCTCATC | 45 | Allen et al. 1995 | 133-135 | 2 | Ned | 6 |
|  |  |  | GAGCCAACTAAGACAAGCC |  |  |  |  |  |  |
|  | GS1 | G02089 | CACATTCTTTTTATGGCTGAATA | 55 | Allen et al. 1995 | 65 | 1 | Ned | 7 |
|  |  |  | AGATGATTGGATAAAGAAGATGTG |  |  |  |  |  |  |
|  | GS2 | G02090 | AATCGAAATGCTGAGCCTCC | 55 | Allen et al. 1995 | 148-154 | 3 | Ned | 8 |
|  |  |  | TGATTTGACTTCCCTTCCCTG |  |  |  |  |  |  |
|  | GS3 | G02091 | TGCACCAGAGCCTAAGCAGACTG | 55 | Allen et al. 1995 | 137-146 | 4 | Fam | 1 |
|  |  |  | CCACCAGCCAGTTCACCCAG |  |  |  |  |  |  |
| *Hydrurga leptonyx* | H12 | AF417692 | **CAAACAATTCAGCACAGACCA*** | 55 | Davis et al. 2002 | 222-239 | 6 | Fam | 3 |
|  |  |  | **TGGAGGATTTGGGAAGTCTG*** |  |  |  |  |  |  |
|  | HL20 | AF140589 | **GCCTGGGTGTCTCTCTCTCA*** | 55 | Gelatt et al. 2001 | 124-126 | 2 | Fam | 4 |
|  |  |  | **TTGACAAGGAGAGTATGTTAACTGG*** |  |  |  |  |  |  |
|  | HL16 | AF140588 | **GCTGGAGAAGCTAGCAGAGG*** | 55 | Gelatt et al. 2001 | 110 | 1 | Fam | 5 |
|  |  |  | **ATCTGCCTTTGGCTTCAGGT*** |  |  |  |  |  |  |
|  | HL15 | AF140587 | **TTGACCCTTCTGTCCCTTTG*** | 55 | Gelatt et al. 2001 | 119-125 | 3 | Fam | 6 |
|  |  |  | **GATCATCTTGTAGTGCCAAAAAC*** |  |  |  |  |  |  |
| *Odobenus rosmarus* | OrrFCB23 | G34939 | ACTGTATGTACAGAGGTTCCCAG | 50 | Buchanan et al. 1998 | 138 | 1 | Fam | 7 |
| *rosmarus* |  |  | CAGTTGTCGACGCTCAGATGTAG |  |  |  |  |  |  |
|  | OrrFCB2 | G34934 | CCATTTCATCCGATGGAAGGAG | 55 | Buchanan et al. 1998 | 109-115 | 4 | Fam | 8 |
|  |  |  | CAAGGACAAGATAGTGACCTAGAC |  |  |  |  |  |  |
|  | OrrFCB1 | G34933 | CTATAACTGGATAGATGATGGTGAC | 60 | Buchanan et al. 1998 | 209-211 | 2 | Fam | 9 |
|  |  |  | CCCAAGCATAAGGTATCTTGGCC |  |  |  |  |  |  |
|  | OrrFCB24 | G34932 | CATCTCCAATCCCTTCTTCCAAC | 50 | Buchanan et al. 1998 | 131-179 | 3 | Hex | 4 |
|  |  |  | TAATTGTTTCTAATGGTCGCTACAAG |  |  |  |  |  |  |


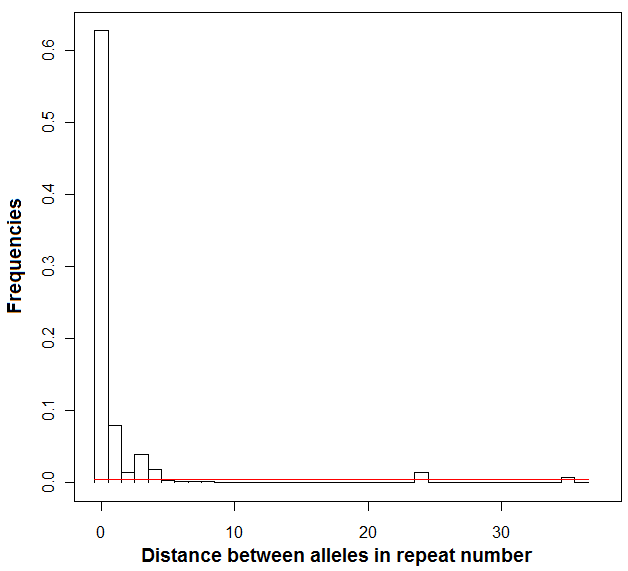


**Appendix C.** Frequencies of distance between the alleles in terms of repeat number (K) on polymorphic (17) microsatellite markers in harbour seal populations from Moray Firth (VAREFF analysis).

**Appendix D.** Pairwise FST below diagonal with value of p-value in italic based on 1,000 bootstraps. Corrected average pairwise difference (PiXY-(PiX+PiY)/2) above the diagonal. Significant p-value (< 0.05) in bold.

|  | Dornoch | Cromarty | Inverness |
| --- | --- | --- | --- |
| Dornoch | - | 0.064 *(0.138)* | 0.058 *(0.196)* |
| Cromarty | 0.014 *(0.135+-0.012)* | - | -0.039 *(0.268)* |
| Inverness | **0.014 *(0.038+-0.005)*** | -0.008 *(0.714+-0.015)* | - |


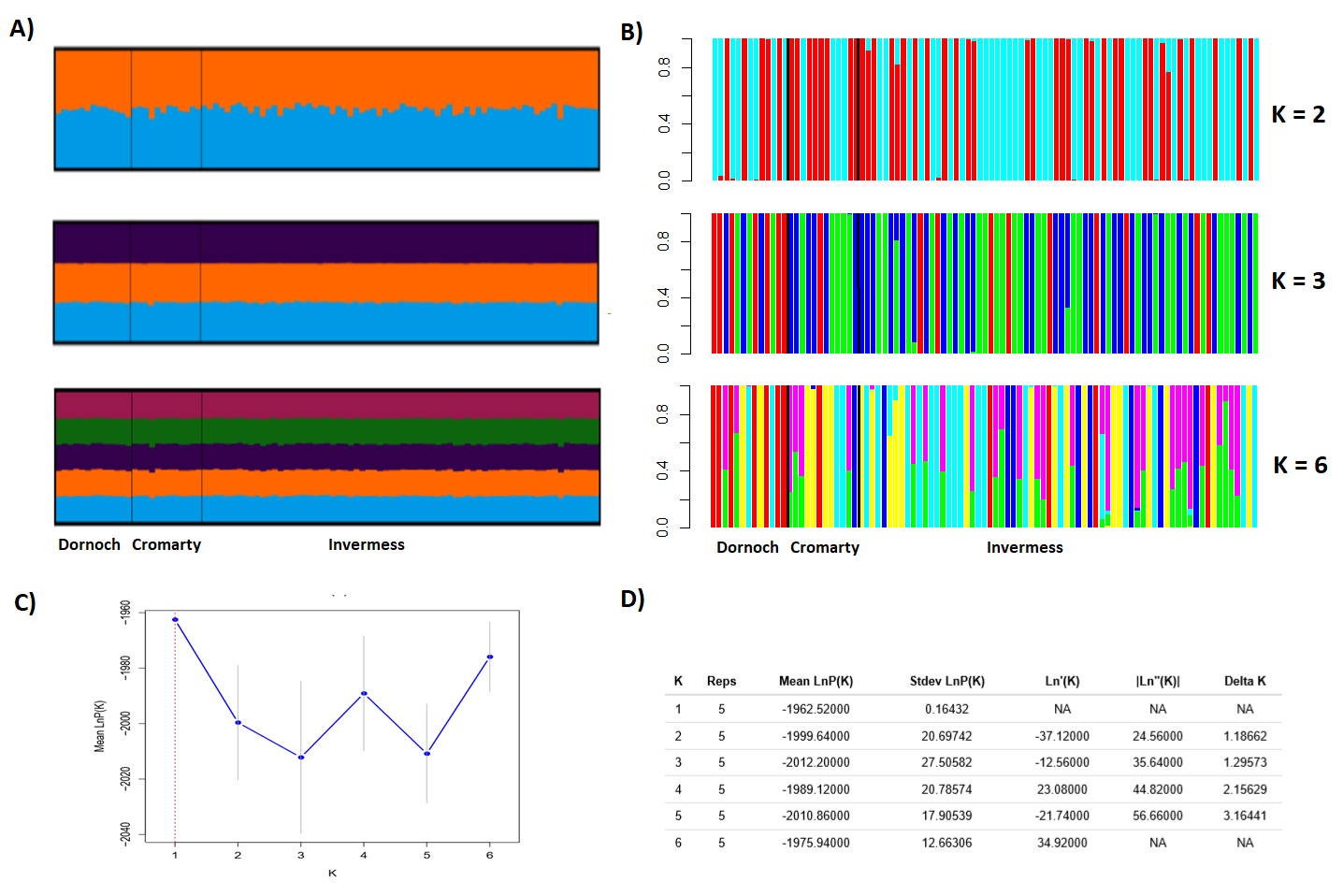


**Appendix E.** Clustering of harbour seals in Moray Firth from STRUCTURE (A) and DAPC (B) analysis for K from 1 to 6 clusters. Each individual is represented by a vertical bar partitioned into coloured sub-bars whose lengths are proportional to its estimated probability of membership for the K clusters, then localisations are on x-axis. Plot of the mean of estimated “log probability of data” for each value of K (C). Evanno table output for K from 1 to 6 (D).

**
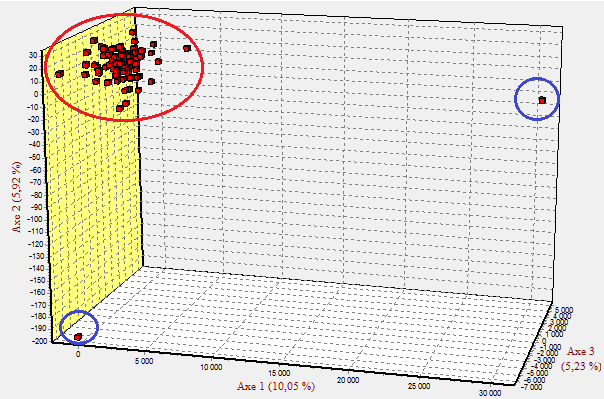
**

**Appendix F.** Factorial correspondence analysis (FCA) in 3 dimensions on individuals of harbour seals in the Moray Firth genotyped with 17 microsatellites. Red circle shows the Moray Firth population, blue circles show the potential immigrants.


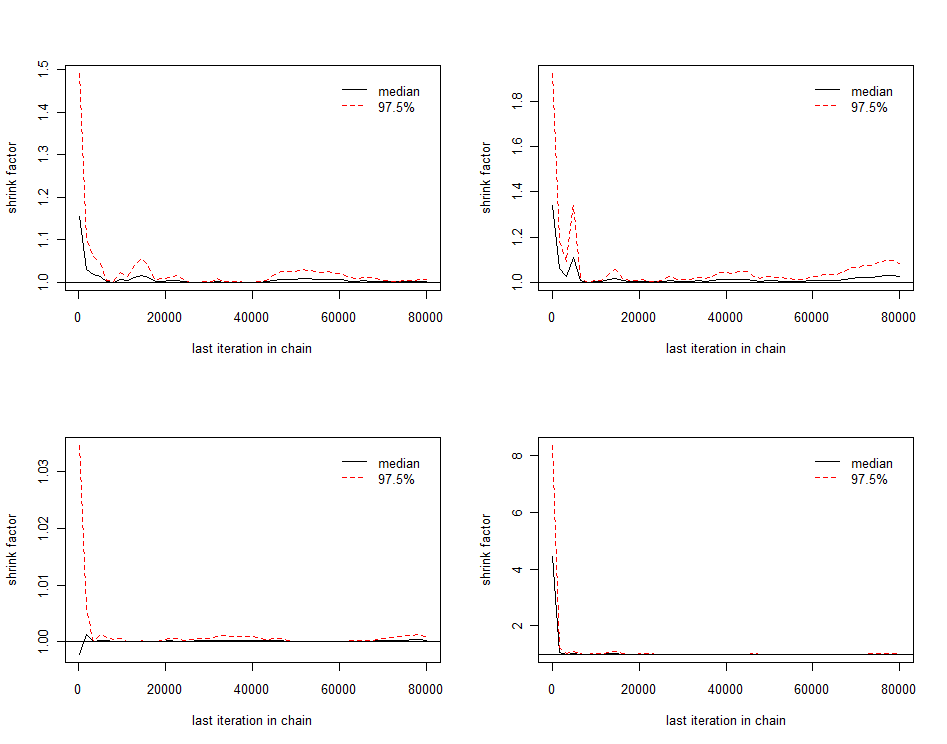


**Appendix G.** Gelman and Rubin's test on four runs with 80,000 chains and 20,000 iterations between chains using MSVAR of harbour seals genotyped with 17 microsatellites markers.

**Appendix H.** Results of the probability of bottleneck detection methods - the sign test, standardized differences test and Wilcoxon test.

| **Test** | **Model** | **Bottleneck** | **Probabilities** |
| --- | --- | --- | --- |
| ***Sign test*** | SMM | Yes | 0.042 |
| ***Standardized differences test*** | SMM | Yes | 0.049 |
| ***Wilcoxon test*** | SMM | Yes | 0.025 |

**Supplementary text**

(**S1**) Genomic DNA was extracted by QIAamp DNA Blood Mini Kit (QIAGEN).

We used the protocol provided by QIAGEN. We describe below the steps of the DNA extraction process.

- Put 20 ul proteinase K in a tube of 1.5 ml.
- Add 200 ul of blood sample.
- Add 200 ul of Buffer AL.
- Mix by vortexing 3 times for 3 seconds each.
- Brief centrifugation with bench-top centrifuge.
- Incubate at 56 ° C for 10 minutes (remember to heat the plates to 56 ° C).
- Brief centrifugation with bench-top centrifuge.
- Add 200 ul of pure ethanol.
- Mix by vortexing 3 times for 3 seconds each = Mix A.
- Prepare the QIAGEN tubes to put the filter in.
- Put the mix (A) inside the QIAGEN with the filter.
- Centrifuge at 8,000 rpm for 1 minute.
- Discard throughflow.
- Add 500 ul of Buffer AW1.
- Centrifuge at 8,000 rpm for 1 minute.
- Discard throughflow.
- Add 500 ul of Buffer AW2.
- Centrifuge at 8,000 rpm for 3 minutes.
- Discard throughflow.
- Place filter into a new QIAGEN collection tube.
- Add 150 ul of Buffer AE.
- Wait 5 minutes
- Centrifuge at 8,000 rpm for 1 minute.
- Add 100 ul of Buffer AE.
- Wait 5 minutes
- Centrifuge at 8,000 rpm for 1 minute.
- What is in the tube is the final concentrated DNA. This DNA was put into sterile Eppendorff tubes.

From this concentrated DNA, we serially diluted samples. For the first dilution, we added 5 μl of this solution with 45 μl of T.E. 1 / 0.1 (Tris 1 and EDTA 0.1) in a new tube. We then assessed DNA concentration using a spectrophotometer. The average of these samples (first diluted - D1 solution) was 60 ug / ml. We wanted our samples at 5ng / ul. We therefore made a second dilution in a new tube by adding 83.5 μl of diluted DNA (Solution D1) and 916.5 μl of T.E.
